# Supplementary material for: Grouping promotes both partnership and rivalry with long memory in direct reciprocity
Source: PLoS Comput Biol. 2023 Jun 20;19(6):e1011228. doi: 10.1371/journal.pcbi.1011228 (PMC10313083; doi:10.1371/journal.pcbi.1011228)
Supplement: S1 Appendix — The simulation results for S(2) and for different parameters are shown. In addition, the model with the full separation of time scale is studied. Fig A. Evolutionary simulations for memory-2 strategy space. Evolutionary simulations for S(2) in a group-structured population (M = 103, N = 2). The upper (a-d) and the lower (e-h) show the results for different out-group selection strengths σout = 30/(b − 1) and σout = 3/(b − 1), respectively. The other simulation parameters are the same as those in Fig 6. Fig B. Simulations with different parameters. Effects of M = 102, to be compared with Fig 6. The other parameters are N = 2, e = 10−6 and σin = 30/(b − 1). For panels (a-h), we have used σout = 30/(b − 1) whereas σout = 3/(b − 1) for the bottom panels. Each simulation runs for 108 time steps, and the results are averaged over 10 independent runs. Fig C. Simulations with completely separated time scales. The simulation results for the case where the timescales for out-group imitations and mutations are completely separated. From left to right, the panels show the cooperation level, (b) the fractions of non-FR efficient strategies, (c) the fractions of non-FR rival strategies, and (d) the fractions of FR strategies. The parameters are same as those in Fig 6a–6f: M = 103, N = 2, e = 10−6, and σin = σout = 30/(b − 1). Simulations were conducted for 106 steps, discarding the initialization period of 105 time steps, and the results were averaged over 10 independent runs. (PDF) [file pcbi.1011228.s001.pdf]

Appendix: Grouping promotes both partnership and rivalry with long memory in direct reciprocity

Yohsuke Murase[1,2*], Seung Ki Baek[3]

1 RIKEN Center for Computational Science, Kobe, Japan
2 Max Planck Research Group 'Dynamics of Social Behavior,' Max Planck Institute for Evolutionary Biology, Plön, Germany
3 Department of Scientific Computing, Pukyong National University, Busan, Korea

* yohsuke.murase@gmail.com

Simulation for memory-2 strategy space

In this section, we show the results of simulations for memory-2 strategy space, $\mathcal{S}(2)$, in a group-structured population. Fig A shows the cooperation levels and the fractions of efficient, rival, and friendly rival strategies, from left to right. If we compare Fig A with Figs. 6e-6l in the main text, the results for $\mathcal{S}(2)$ are qualitatively similar to those for $\mathcal{S}(3)$, indicating that the dynamics of the group-structured population is robust as long as the strategy space contains FRs.

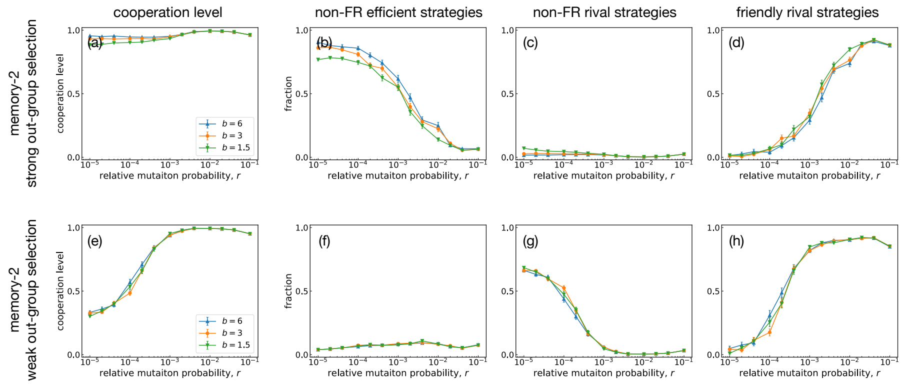

Fig A. Evolutionary simulations for memory-2 strategy space. Evolutionary simulations for $\mathcal{S}(2)$ in a group-structured population ($M = 10^3, N = 2$). The upper (a-d) and the lower (e-h) show the results for different out-group selection strengths $\sigma_{\text{out}} = 30/(b-1)$ and $\sigma_{\text{out}} = 3/(b-1)$, respectively. The other simulation parameters are the same as those in Fig 6.

Simulation with different parameters

To test the robustness of our simulation results, we have conducted simulations with different parameters. Fig B shows results for $M = 10^2$. These results are qualitatively similar to those with $M = 10^3$ in Fig. 6 in the main text. The main difference from Fig. 6 is the dependency on the relative mutation probability r in the memory-three strategy space: For $M = 10^3$, non-FR strategies make way for FRs at $r \approx 10^{-3}$,

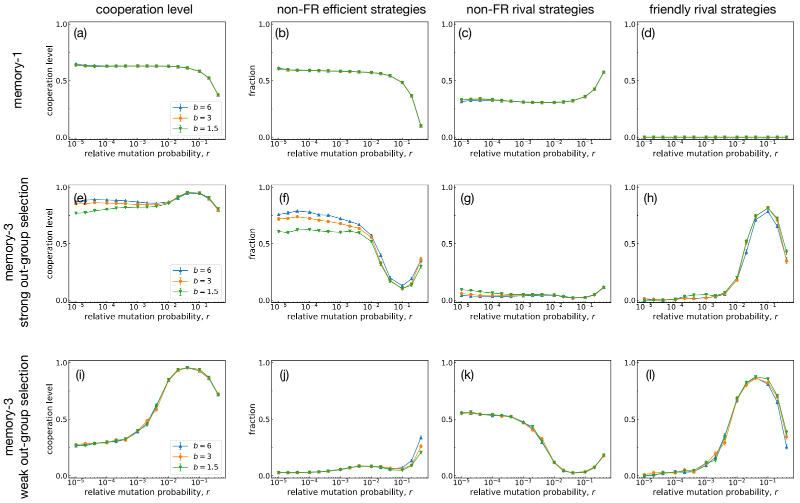

Fig B. Simulations with different parameters. Effects of $M = 10^2$, to be compared with Fig 6. The other parameters are $N = 2$, $e = 10^{-6}$ and $\sigma_{\text{in}} = 30/(b-1)$. For panels (a-h), we have used $\sigma_{\text{out}} = 30/(b-1)$ whereas $\sigma_{\text{out}} = 3/(b-1)$ for the bottom panels. Each simulation runs for 10^8 time steps, and the results are averaged over 10 independent runs.

whereas a similar transition occurs at $r \approx 10^{-2}$ when $M = 10^2$. To see why, let us recall that the biggest threat to FRs is the neutral drift caused by efficient strategies. For FRs to survive long, therefore, r needs to be high enough, i.e., $\gtrsim O(1/M)$, to suppress non-FR efficient strategies. If r is even higher, the dynamics is mostly driven by mutation while out-group imitation hardly occurs. As a result, the results becomes similar to those from a well-mixed population with $N = 2$.

We have also investigated the evolution of average memory lengths in these simulations and found similar results to the ones in Fig. 9.

Full separation of time scales

In the main text, we have considered the case where the time scale for out-group imitation is comparable with that of mutation. Here, for the sake of completeness, we study the case where the time scales are completely separated, i.e., by setting $\nu \ll \mu_{\text{out}} \ll \mu_{\text{in}}$, so that mutation occurs far less frequently than the other processes. Again, once a mutant is introduced, no other mutation occurs until the mutant takes over the population or dies out. The fixation probability that a mutant Y takes over the population with strategy X is

$$\Psi_{X \to Y} = \rho_{X \to Y} \frac{1}{1 + \sum_{j=1}^{M} \eta^j}, \tag{1}$$

where $\eta \equiv T_{Y \to X}/T_{X \to Y}$ [1].

Monte Carlo simulations are conducted in the same way as explained in Methods above, and the parameter values are $M = 10^3$, $N = 2$, $e = 10^{-6}$, and $\sigma_{\text{in}} = \sigma_{\text{out}} = 30/(b-1)$. Fig C shows the results. In $\mathcal{S}(1)$, the cooperation level strongly depends on b, the benefit of cooperation: Cooperation level is almost 100% for $b = 6$, but it is less than a half at $b = 3$ and accompanied by a proliferation of rivals. This is consistent with our previous study [1]. When $\mathcal{S}(3)$ is available, the cooperation level is less sensitive to b and actually higher than in $\mathcal{S}(1)$ except for $b = 6$. The first reason is

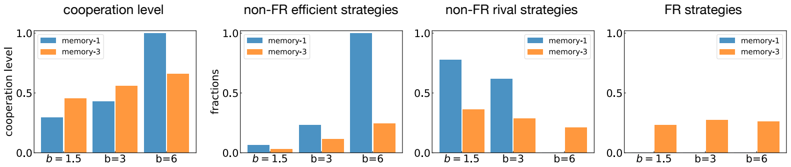

Fig C. Simulations with completely separated time scales. The simulation results for the case where the timescales for out-group imitations and mutations are completely separated. From left to right, the panels show the cooperation level, (b) the fractions of non-FR efficient strategies, (c) the fractions of non-FR rival strategies, and (d) the fractions of FR strategies. The parameters are same as those in Fig 6a-6f: $M = 10^3$, $N = 2$, $e = 10^{-6}$, and $\sigma_{\text{in}} = \sigma_{\text{out}} = 30/(b-1)$. Simulations were conducted for 10^6 steps, discarding the initialization period of 10^5 time steps, and the results were averaged over 10 independent runs.

that the cooperation level for $b = 6$ in $\mathcal{S}(1)$ is unusually high because of the absence of the dangerous mutants that can threaten WSLS in $\mathcal{S}(1)$. Namely, WSLS is stable against mutants in $\mathcal{S}(1)$ due to their poor performance, but it is no longer stable against the mutants in $\mathcal{S}(3)$. The second and more important reason is that FRs contribute to increasing the cooperation level even for $b = 1.5$ and $b = 3$ as shown in the right panel of Fig C. We point out that the fraction of FRs does not reach 100% in this setting because they still suffer from the neutral drift due to non-FR efficient strategies when $r \to 0$.

One technical point that should be noted here is that it is not easy to observe a crossover from the partial to the full separation of time scales by simulation. This is because the time scales for population-wide fixation are often exceedingly long in group-structured populations [1]. While efficient strategies are selected by out-group imitation, they fail to take over the group again and again, thus leading to a long time scale for the fixation of a mutant. In this sense, the partial separation of time scales studied in the main text is a more reasonable assumption than the full separation.

References

1. Murase Y, Hilbe C, Baek SK. Evolution of direct reciprocity in group-structured populations. Scientific Reports. 2022;12(1):1–16.
