# Supplementary material for: Grouping promotes both partnership and rivalry with long memory in direct reciprocity
Source: PLoS Comput Biol. 2023 Jun 20;19(6):e1011228. doi: 10.1371/journal.pcbi.1011228 (PMC10313083; doi:10.1371/journal.pcbi.1011228)
Supplement: Attachment — Submitted filename: reply.pdf [file pcbi.1011228.s002.pdf]

Max Planck Institute for Evolutionary Biology
Plön, Germany
YOHSUKE MURASE
Email: yohsuke.murase@gmail.com

April 26, 2023

Dear Editor,

We are grateful for your consideration of our manuscript entitled "Grouping promotes both partnership and rivalry with long memory in direct reciprocity." We are pleased that all three reviewers evaluated our work quite positively such as *"the authors successfully obtained some new findings, which can be highly evaluated and quite informative," "the authors do an excellent job,"* and *"a fascinating paper."* Their careful reading and constructive suggestions are greatly appreciated, and we have tried our best to answer their comments and questions as detailed below. We have also made typesetting corrections and minor revisions to improve the clarity of our manuscript. We hope that this revised manuscript is now acceptable for publication in Plos Computational Biology.

Yours Sincerely,

Yohsuke Murase
on behalf of the authors

To Reviewer 1

> **Reviewer 1:** By going thru a stringent MAS (by taking Monte Caro simulation) approach with support of some theoretical underline, the authors successfully obtained some new findings, which can be highly evaluated and quite informative to be shared to the audience in the scientific arena. Scientific health and solidness seem reliable. Hence, I would like to recommend this work to be published.

Answer: First of all, we are grateful for your careful reading and constructive suggestions. We have revised our manuscript in accordance with your report.

> **Reviewer 1:** Just one technical suggestion to add impressiveness is given as below. As I above mentioned, the game structure the authors presumed belong to D & R game, one of sub-classes of PD, which is a standard template for PD theoretical biologists have heavily favored. That's fine. In usual definition there are two parameters; b and c. They presumed c=1 unity, which is perfectly ok. But they should carefully explain it, and should mention that the dilemma strength in such case; measured by the universal dilemma strength by Dg' and Dr', is parameterized by a single parameter; b (more precisely; 1/(b-1)). The additional part should be added to Model depiction, which should be accompanied by citation and review on relevant literatures, for instance, (i) Relationship between dilemma occurrence and the existence of a weakly dominant strategy in a two-player symmetric game, BioSystems 90(1), 105-114, 2007, (ii) Universal scaling for the dilemma strength in evolutionary games, Physics of Life Reviews 14, 1-30, 2015, (iii) Scaling the phase- planes of social dilemma strengths shows game-class changes in the five rules governing the evolution of cooperation, Royal Society Open Science, 181085, 2018, (iv) Sociophysics Approach to Epidemics, Springer, 2021.

Answer: Thank you for your suggestions. We revised our manuscript to clarify that the dilemma strength is characterized by a single parameter b. We added the suggested citations, with which our manuscript gets even clearer. Please see the changes in Model section, line 120.

To Reviewer 2

> **Reviewer 2:** I really enjoyed reading this paper. The authors do an excellent job of presenting the background and motivation for their study. While I do have some comments below, I think this paper should be published in PLOS Computational Biology after some minor revisions.

Answer: We appreciate your efforts in reading our manuscript and the constructive suggestions. We are so happy to hear that you enjoyed reading our manuscript and evaluated it in such a positive way. The followings are our point-by-point responses to your comments.

> **Reviewer 2:** Abstract: remove "large-scale"

Answer: We revised the abstract accordingly.

> **Reviewer 2:** Abstract: it's not clear why there is a jump from memory-one to memory-three (why not memory-two, a reader might ask?)

Answer: I agree with you that the readers might get curious about why memory-three is used. The reason why we used memory-three is memory-three is the longest memory length we can practically handle in our simulations, and we intended to highlight the difference between memory-one and beyond. We indeed studied memory-two, but we found that memory-two is not so different from memory-three. We added the results for memory-two in Appendix, and in the abstract, we rewrite "memory.-three" to "longer-memory". We also added a sentence why we focus on memory-three in the main text. See line 108 in p4.

> **Reviewer 2:** Introduction, paragraph two: I would not say that it reduces a two-body problem to a one-body problem, since this could be said of any play in which one agent fixes a strategy and allows the other to choose. Instead, as other ZD studies have stated, it would be more descriptive to say that it induces an ultimatum on the other player.

Answer: Thank you for your suggestion. We agree with you and revised the sentence accordingly. See line 30 in the revised manuscript.

> **Reviewer 2:** Introduction, paragraph four: When mentioning CAPRI, you should discuss briefly the finding that if a player uses a memory-one strategy then they can assume WLOG that the opponent also uses a memory-one strategy. At first, it seems confusing that a longer-memory strategy could outperform a memory-one strategy, but in the background, I assume what the authors are referring to is the (population-based) performance of memory-k versus memory-k for $k > 1$?

Answer: Thank you for your question. What we consider here is the performance in evolutionary simulation (namely, the population-based performance). As you said, CAPRI (or other longer memory strategies) behaves like memory-one when the opponent's strategy is memory-one. So no strategy (including longer-memory ones) can outperform a rival strategy in a one-by-one game. However, FR strategies can recover from erroneous moves, by making use of longer strategies. While CAPRI forms full cooperation against other CAPRI players, it behaves like Grim Trigger against most other strategies. This is the reason why CAPRI is strong in evolutionary simulations. I added a sentence in the introduction to make it clear that we consider population-based performance. See line 67 in Introduction.

> **Reviewer 2:** Figure 1: change the shading of the feasible region to another color or change the blue bubble above to something else. It is confusing when the colors are referred to in the text because they look the same to the naked eye (although maybe they are slightly different shades?).

Answer: Thank you for the nice suggestion. We changed the color of the shaded areas for clarity.

> **Reviewer 2:** Introduction, final paragraph: remove "large-scale"

Answer: We revised it accordingly.

> **Reviewer 2:** Introduction, final paragraph: oh, so you mean boundary strategies within the memory-k (k in 1,3) space, since you state that they have finite cardinality?

Answer: In this paper, we consider only pure strategies. We revised line 75 and line 107 in order to make this more explicit.

Actually, FR strategies must have deterministic moves for some of their prescriptions, and FRs have measure zero in the mixed-strategy space. We expected that the strategies close to FR strategies evolve in mixed-strategy spaces, but how to characterize the closeness to FR is another challenge. We leave it for future work, and we discussed this possibility in Discussion. Please see a paragraph at line 401.

> **Reviewer 2:** Model, paragraph one: is the purpose of implementation errors just to ensure that the Markov chain is ergodic?

Answer: While implementation errors ensure ergodicity, it is not the primary reason. Implementation errors are introduced to make the model more realistic as commonly assumed in previous studies.

> **Reviewer 2:** Before equations (3) and (4), π_X and π_Y need to be defined. In a finite group, does an individual interact with everyone and average those payoffs to get π_X and π_Y?

Answer: Thank you for pointing this out. As you said, π_X and π_Y are the average payoffs after the interaction with everyone else in the group. We revised the manuscript accordingly. See Eq.(3) in the revised manuscript.

> **Reviewer 2:** On line 198, replace "naively" with "uniformly"

Answer: We revised it accordingly.

> **Reviewer 2:** Remove "significant" from line 203 to avoid confusion with statistical significance.

Answer: Thank you for your suggestion. We used "non-negligible" instead.

> **Reviewer 2:** Right at the beginning of "Results" the figure is referred to for a qualitative comparison between memory-1 and memory-3 strategies. What is the "fraction" that appears there? Is this a stationary fraction after many steps? Or up to some finite generation?

Answer: The fraction is the stationary fraction after a sufficiently long initial period (10^5). We revised the figure caption accordingly.

> **Reviewer 2:** On line 231, remove "Nash" since a SPE is a refinement of a Nash equilibrium.

Answer: We revised it accordingly. Thank you.

> **Reviewer 2:** On line 237, what is an example of a "dangerous mutant" from $S(3)$ that can threaten WSLS? Is there an intuitive reason for why WSLS is susceptible strategies with longer memory capacities?

Answer: Thank you for your sharp question. These "dangerous mutants" are the mutants that have a strictly higher payoff than WSLS while they have high self-cooperation levels. Such strategies are able to invade WSLS, but happen to be absent in $S(1)$. We revised the manuscript to make this point clearer. See line 255.

> **Reviewer 2:** When talking about "evolution of memory lengths," the authors mention that $m_2 > m_1$ is favored, meaning players care more about having a longer memory of the opponent than of themselves. Could one reason for this be the fact that the central game considered is a donation game (which is additive)? Would the same qualitative findings hold for the non-additive PD with $(R, S, T, P) = (3, 0, 5, 1)$?

Answer: Thank you for another nice question. Although we assumed the donation game in this study, we do not think the results are specific to the donation game. Whether a strategy is a friendly rival or not is independent of the values of R, S, T, P as long as mutual cooperation is socially optimal $2R > T + S$. Therefore, we consider that the results for the group-structured population are also insensitive to these values. We added a paragraph to discuss this point in the revised manuscript. See the paragraph at line 386 in Discussion.

> **Reviewer 2:** One weakness of this study, which the authors mention indirectly toward the end, is that it relies on imitation with mutation. Since strategies here are not binary actions which can be easily observed, the ability to imitate a strategy presupposes that these strategies can be observed. I don't really think that just raising the mutation rate accounts for this, at least in terms of human behavior. Mutation rates at the level of the conditional strategy are more natural in non-cultural settings like birth-death processes. If one were to take a group-structured population with BD updating a migration, would you observe similar results? I am not suggesting the authors add a lot of new material on BD updating, but it would be really helpful for the paper if this could be checked in some level of depth and at least commented on in the paper.

Answer: Thank you for your insightful comments. We agree with you that the "imitation" of longer-memory strategies is not necessarily easy and it might be more reasonable to assume a "biological" process rather than a "cultural" process. Although we do not have a strong opinion on which is more realistic, we consider that our findings in this manuscript are robust for other strategy updating models. This is because the key mechanism behind our results is the fact that friendly rivals are strong both in in-group and out-group interactions. Therefore, we added a paragraph to discuss this point in the revised manuscript. Again, see the paragraph at line 386 in Discussion.

To Reviewer 3

> **Reviewer 3:** This is a fascinating paper and I support publication. I have two comments that the authors might wish to address in a revision

Answer: We really appreciate your comments and suggestions. We revised the manuscript accordingly. Please see our responses below. While your summary catches the point of our work, one point we would like to correct is that our work is not about n=3 public goods game but the Prisoner's Dilemma. Perhaps, Fig.2 and our focus on memory-three strategies might have confused you. We answered why we chose $m = 3$ in the main text (line 108) and added some results for memory-2 to Appendix. We believe that the current manuscript got clearer.

> **Reviewer 3:** 1) The authors study the limit in which in-group imitation is much faster than both out-group imitation and mutation. They then study the impact of the parameter $r = \nu/(\mu_{out} + \nu)$ on the evolutionary dynamics (i.e. the relative rate of mutation to out-group imitation). This generates a kind of weak-mutation limit at the group level (each group will be composed of at most two strategies at any given time). In the Appendix they also study a full separation of timescales in which mutation is truly weak (i.e. at most two strategies exist in the population at any one time, and in-group imitation is fast compared to out-group). These are both interesting limits but it is not clear to me that $\mu_{out} <$

Answer: We're glad to hear that you find the results for both limits interesting. Your last sentence is not clear to us as your comment is cut off in the middle for some reason. We guess your question might be "Do these two limits converge when $r \to 0$?" If so, the answer is yes although r must be exponentially small to see the convergence. As discussed in our previous paper [Murase,Hilbe,Baek Sci.Rep.(2022)], typical time scales to reach the system-wide fixation are often exponentially long in the group-structured population. Therefore, we consider that the partial time-scale separation, studied in the main text, is a more reasonable assumption than the full separation of timescales. We added a paragraph in Appendix to discuss this point in the revised manuscript. Please see the paragraph at line 648 in Appendix.

> **Reviewer 3:** 2) In the simulations it's not entirely clear to me whether only pure strategies are considered. In Figure 3 and on page 7 pure strategies are discussed but in Figure 1 strategies such as extortion are discussed which use cooperation probabilities other than 0 or 1. This choice matters because the results in well-mixed populations are driven by the relative rarity of FR strategies. Do FR strategies become more or less rare when we move away from pure strategies, and how does that impact the results in group structured populations?

Answer: Thank you for your question. This is a similar question given by Reviewer 2, and we agree that our manuscript was not clear enough. In this paper, we consider only pure strategies. We made it more explicit that we consider only pure strategies in the revised Introduction. See line 76 and 107.

Indeed, mixed strategies would be an interesting topic for future studies. In the mixed strategy space, the fraction of FR strategies tends to zero as some of the prescriptions must be deterministic. For instance, cooperation and defection must be prescribed for sure during mutual cooperation and defection, respectively. However, we surmise that the overall results remain the same even with mixed strategies. We consider that the strategies that are close to FR strategies will appear even though how to define the "closeness" remains another challenge for future studies. We added a paragraph to discuss this issue in Discussion. See the paragraph starting at line 401.

> **Reviewer 3:** 3) In the evolution of memory section (Figure 9), how are mutations that change memory length implemented? That is, do we just assume that any strategy of any memory length can mutate to any other? Or is there some sense in which mutations are local (eg memory can only increase by one unit at a time)?

Answer: This is another nice question. In our study, any memory length can mutate to any other. As we mentioned in "Memory lengths of strategies" section, memory lengths are randomly uniformly drawn for each mutation event. However, we agree with you that it is more reasonable to assume that memory lengths (and strategy) could be gradually changed. We added a paragraph to discuss this possible model extension for future studies in Discussion. See the paragraph starting at line 401.
